# Neonatal Screening for Congenital Adrenal Hyperplasia in Turkey: A Pilot Study with 38,935 Infants

**DOI:** 10.4274/jcrpe.galenos.2018.2018.0117

**Published:** 2019-02-20

**Authors:** Tülay Güran, Başak Tezel, Fatih Gürbüz, Beray Selver Eklioğlu, Nihal Hatipoğlu, Cengiz Kara, Enver Şimşek, Filiz Mine Çizmecioğlu, Alev Ozon, Firdevs Baş, Murat Aydın, Feyza Darendeliler

**Affiliations:** 1 Marmara University Faculty of Medicine, Department of Paediatric Endocrinology and Diabetes, İstanbul, Turkey; 2 Turkish Directorate of Public Health, Ankara, Turkey; 3 Çukurova University Faculty of Medicine, Department of Paediatric Endocrinology and Diabetes, Adana, Turkey; 4 Necmettin Erbakan University Meram Faculty of Medicine, Department of Paediatric Endocrinology and Diabetes, Konya, Turkey; 5 Erciyes University Faculty of Medicine, Department of Paediatric Endocrinology and Diabetes, Kayseri, Turkey; 6 Ondokuz Mayıs University Faculty of Medicine, Department of Paediatric Endocrinology and Diabetes, Samsun, Turkey; 7 Osmangazi University Faculty of Medicine, Department of Paediatric Endocrinology and Diabetes, Eskişehir, Turkey; 8 Kocaeli University Faculty of Medicine, Department of Paediatric Endocrinology and Diabetes, Kocaeli, Turkey; 9 Hacettepe University Faculty of Medicine, Department of Paediatric Endocrinology and Diabetes, Ankara, Turkey; 10 İstanbul University İstanbul Faculty of Medicine, Department of Paediatric Endocrinology and Diabetes, İstanbul, Turkey

**Keywords:** Newborn screening, congenital adrenal hyperplasia, second-tier, steroid profiling

## Abstract

**Objective:**

Congenital adrenal hyperplasia (CAH) is the most common form of primary adrenal insufficiency in children. Neonatal screening for CAH is effective in detecting the salt-wasting (SW) form and in reducing mortality. In this study, our aim was to estimate the incidence of CAH in Turkey and to assess the characteristics and efficacy of the adopted newborn CAH screening strategy.

**Methods:**

A pilot newborn CAH screening study was carried out under the authority of the Turkish Directorate of Public Health. Newborn babies of ≥32 gestational weeks and ≥1500 gr birth weight from four cities, born between March 27-September 15, 2017 were included in the study. Screening protocol included one sample two-tier testing. In the first step, 17α-hydroxyprogesterone (17-OHP) was measured by fluoroimmunoassay in dried blood spots (DBS) obtained at 3-5 days of life. The cases with positive initial screening were tested by steroid profiling in DBS using a liquid chromatography-tandem mass spectrometry method to measure 17-OHP, 21-deoxycortisol (21-S), cortisol (F), 11-deoxycortisol and androstenedione as a second-tier test. The babies with a steroid ratio (21-S+17-OHP)/F of ≥0.5 were referred to pediatric endocrinology clinics for diagnostic assessment.

**Results:**

38,935 infants were tested, 2265 (5.82%) required second-tier testing and 212 (0.54%) were referred for clinical assessment, six of whom were diagnosed with CAH (four males, two females). Four cases were identified as SW 21-hydroxylase deficiency (21-OHD) (two males, two females). One male baby had simple virilizing 21-OHD and one male baby had 11-OHD CAH. The incidence of classical 21-OHD in the screened population was 1:7,787.

**Conclusion:**

The incidence of CAH due to classical 21-OHD is higher in Turkey compared to previous reports. We, therefore, suggest that CAH be added to the newborn screening panel in Turkey. The use of steroid profiling as a second-tier test was found to improve the efficacy of the screening and reduce the number of false-positives.

**What is already known on this topic?**

Classical congenital adrenal hyperplasia (CAH) occurs in 1:13,000 to 1:15,000 live births. 21-hydroxylase enzyme deficiency (21-OHD) occurs in 90 to 95% of all cases of CAH. CAH is a potentially life-threatening condition that requires accurate diagnosis and urgent treatment with glucocorticoid and mineralocorticoid replacement. Neonatal screening for CAH is effective in detecting the salt-wasting form and thereby reducing mortality.

**What this study adds?**

The estimated incidence of classical 21-hydroxylase enzyme deficiency (21-OHD) congenital adrenal hyperplasia (CAH) in the screened population in Turkey was 1:7,787. The incidence of CAH due to classical 21-OHD is higher in Turkey in comparison to previous reports in the literature. Thus, it may be worthwhile to add CAH to the newborn screening panel in Turkey.

## Introduction

Congenital adrenal hyperplasias (CAH) arise from biallelic gene defects encoding the enzymes and cofactor proteins involved in cortisol (F) biosynthesis. The most common enzyme deficiency that accounts for more than 90% of all cases with CAH is 21-hydroxylase deficiency (21-OHD). 21-OHD is classified into three subtypes according to clinical severity: classical salt wasting (SW), classical simple virilizing (SV) and nonclassical CAH (NCCAH; mild or late onset) (1). Data from nearly 6.5 million newborn screenings (NBS) worldwide indicate that classical CAH occurs in 1:13,000 to 1:15,000 live births (2).

CAH is the most common cause of primary adrenal insufficiency in childhood and is a potentially life-threatening condition that requires accurate diagnosis and urgent treatment with glucocorticoid and mineralocorticoid replacement. Symptoms and signs may easily be overlooked, particularly in male infants who do not have genital ambiguity. Because of delayed or missed diagnosis in affected male infants (and some very virilized female infants), in 2002 the Joint Lawson Wilkins Paediatric Endocrine Society/European Society for Pediatric Endocrinology Working Group recommended biochemical screening for CAH in the newborn period (3,4). The majority of states in the United States (US) and more than 50 countries are currently performing NBS for CAH (5). Infant screening programs have markedly decreased the time to diagnosis, theoretically decreasing morbidity (6,7). Based on proven importance, a pilot NBS programme for CAH was initiated by the Turkish Directorate of Public Health (TDPH) on March 27, 2017 in four Turkish cities. We have evaluated the data collected from this pilot study to describe the incidence of CAH in Turkey. We have also described the cases with CAH identified by this pilot study in detail. Additionally, we assessed the results of the pilot study in detail with regard to the characteristics and efficacy of the adopted NBS strategy to determine if any modifications to the strategy would enhance screening performance.

## Methods

**Figure 1:**
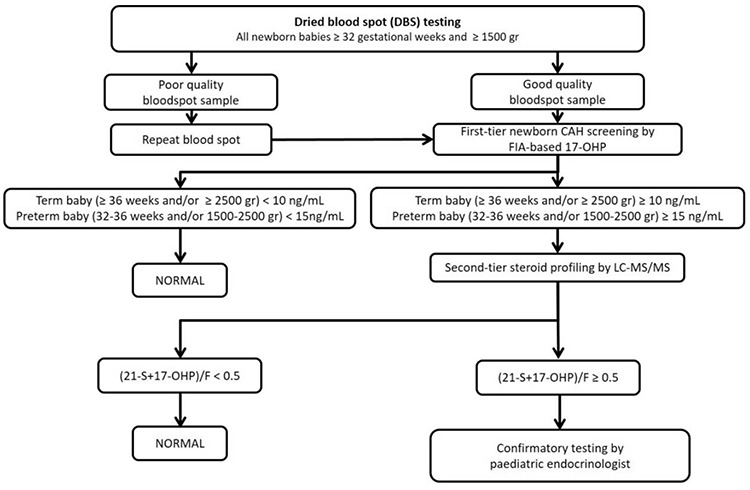
Flowchart for pilot neonatal congenital adrenal hyperplasia screening initiated by the Turkish Directorate of Public Health CAH: congenital adrenal hyperplasia, FIA: fluoroimmunoassay, 17-OHP: 17-hydroxyprogesterone, LC-MS/MS: liquid chromatography-tandem mass spectrometry, F: cortisol

The pilot screening programme for CAH was carried out between March 27 and July 15, 2017 by the TDPH, in four Turkish cities (Adana, Kayseri, Konya and Samsun). According to the programme, dried blood spots (DBS) were obtained using filter paper (“Guthrie” cards) between the 3^rd^ and 5^th^ day of life or as soon as possible after 48 hours of age, by heel prick. The samples were obtained simultaneously with the ongoing nationwide NBS program for congenital hypothyroidism, phenylketonuria, biotinidase deficiency and cystic fibrosis. The CAH screening algorithm was developed in consultation with an expert scientific committee, consisting of paediatric endocrinologists from several universities in Turkey (Figure 1). Newborn babies ≥32 gestational weeks (gw) and ≥1500 gr birth weight from the four cities where the pilot study was conducted were included. 

Initial CAH screening was based on the measurement of 17α-hydroxyprogesterone (17-OHP) in DBS on filter paper by fluoroimmunoassay (FIA) (Labsystems Diagnostics, Finland). Cut-off values for 17-OHP were based primarily on gestational age and birth weight. 17-OHP values of 10 ng/mL and 15 ng/mL have been used as cut-off points for newborn babies ≥36 gw and/or ≥2500 gr birth weight and for newborn babies between 32-36 gw and/or 1500-2500 gr birth weight, respectively (8,9,10). If the 17-OHP level was above the cut-off level in the first-tier test using immunoassay, the filter paper was directly analyzed by liquid chromatography-tandem mass spectrometry (LC-MS/MS) for a steroid profiling assay for simultaneous analysis of 17-OHP, 21-deoxycortisol (21-S), F, androstenedione (4AS) and 11-deoxycortisol (11-S). Normal values for babies of 32-36 gw and/or 1500-2500 gr were; 17-OHP: <8 ng/mL, 21-S: <1.5 ng/mL, F: >50 ng/mL, 4AS: <4.5 ng/mL. Normal ranges for babies ≥36 weeks and/or ≥2500 gr were; 17-OHP: <1.5 ng/mL, 21-S: <1.5 ng/mL, F: > 50 ng/mL, 4AS: <4.5 ng/mL. Although all of the steroids were evaluated for each baby; a (21-S+17-OHP)/F ratio of ≥0.5 was considered as the main criterion for referral (Figure 1) (11,12,13).

## Reagent, Instruments, and Analytical Conditions for Liquid Chromatography-tandem Mass Spectrometry

The steroid standards for F, 17-OHP, 21-S, 11-S, 4AS and deuterated steroid standards for d4-F, d8-17-OHP, d3-testosterone were purchased from Sigma-Aldrich (MO, USA). Acetonitrile (ACN), methanol, ethanol, isopropyl alcohol (IPA), formic acid and LC-MS grade water were purchased from Merck (Darmstadt, Germany).

Detection and measurement were performed on a QTRAP^®^ 5500 tandem MS equipped with an Sciex Exion AC LC system (AB Sciex, Concord, Ontario, Canada) that was operated using an electrospray ionization source in positive and multiple reactions monitoring mode. The column used was Phenomenex Kinetex C18, 100 mm Å~2.1 mm, 2.7 µ (Phenomenex, Torrance, CA, USA) that was maintained at 50 °C. The mobile phase gradient conditions consisted of water (A) (containing 0.1% v/v Formic Acid in Water) and ACN (B) (containing 0.1% v/v Formic Acid in ACN). The flow rate was 0.35 mL/min and the final injection volume of each sample was 20 µL. All sample extracts were maintained in the autosampler at 4 °C while awaiting injection. The ionization source conditions were as follows: curtain gas: 25 psi; ion spray voltage: 5500V; temperature: 500 °C; nebulizer gas (GS1): 50 psi and heater gas (GS2): 50 psi. The optimized precursor and product ion pairs, collision energy and retention times for the analytes and internal standards are listed in Table 1.

### Sample Preparation

In order to obtain calibrators and control DBS, blood from a healthy donor was washed four times with saline to remove all plasma. The washed cells were then combined with steroid-free serum in proportions that resulted in a hematocrit of 0.50. A mix of unlabeled steroid hormones stock solutions of 1 mg/mL in ethanol was diluted in steroid-free serum to obtain four points for calibration and two points for control. F concentrations were 0, 4.12, 37.0, 333.33 nmol/L in calibrators and 12.3 and 111.1 nmol/L in controls. All other analyte concentrations were 0, 2.1, 18.5, 166.7 nmol/L in calibrators and 6.2 and 55.6 nmol/L in controls. Internal standard stock solutions of 1 mg/mL were prepared in ethanol for all deuterium-labeled steroid hormones and diluted to 40 mmol/L in IPA/ACN.

The blood spots, each 4x3 mm in diameter were punched out of each DBS calibrator, control and sample using a manual puncher into a tube and 500 uL of internal standard mix was added. The tubes were mixed for 60 min by an orbital shaker. Supernatant was transferred to a 96-well plate and evaporated at 50 °C by vacuum centrifuge. 50 uL of a methanol/water mixture was added to reconstitute the dry residues and 20 uL injected by limited insert vials.

### Ethics

The parents were informed about NBS. Heel-prick blood samples were collected from live-born babies after written consents from the parents were obtained. The study was carried out with the written permission of the Scientific Committee of the TDPH.

### Statistical Analysis

Statistical evaluation was performed using GraphPad Prism^®^ V5.0 software (GraphPad Software Inc., San Diego, California, USA). The results for each steroid are reported as mean, standard deviation (SD) or as median in the text. We performed a *t-test* for the comparison of the means of two independent samples. Values were considered statistically significant when p value was less than 0.05.

## Results

The total number of newborns that underwent CAH screening was 38,935. Of those babies, 33,967 (87.2%) were ≥36 gw and ≥2500 gr birth weight. There were 3,022 babies (7.8%) between 1500-2500 gr birthweight and 3,684 babies (9.5%) born between 32-36 gw. 1,744 (4.5%) babies were born between 32-36 gw and had a birthweight of 1500-2500 gr. 

Results of first-tier 17-OHP measurement using DBS of the normal newborn population (those without CAH) are summarized in Table 2. We have presented 99.8 and 99.5% of 17-OHP for healthy babies to define healthy cut-off values with a greater sensitivity (14).

2,265 (5.8%) babies had second-tier testing by LC-MS/MS steroid profiling using the same DBS. During screening the babies born between 32-36 gw and/or of 1500-2500 gr birthweight were more likely to fail to pass first-tier and a much higher proportion in these categories required second-tier testing in comparison to those with a birthweight of ≥2500 gr and/or a gestational age ≥36 weeks (Table 3). 

Two hundred and twelve babies who failed to pass second-tier testing were referred to paediatric endocrinology clinics for further evaluation, which corresponds to an overall recall rate of 0.54%.

Table 4 shows the distribution of second-tier testing values of babies referred for further analysis. The results are summarized with respect to gestational age and birth weight. The highest proportion of the babies referred to clinics had a (21-S+17-OHP)/F ratio between 0.5-1. 

The babies referred to paediatric endocrinology clinics were evaluated by medical history and physical examination for CAH symptoms and signs. Serum electrolytes were measured and in most of the babies 17-OHP testing was repeated, mainly by LC-MS/MS or immunoassay. Based on this evaluation, further biochemical assessments including synacthen test, ACTH, renin and detailed plasma steroid measurements by LC-MS/MS were undertaken when necessary and only for the cases suggestive of CAH. Genetic testing was performed only if the diagnosis of CAH was established by clinical and biochemical findings. Molecular analysis of the *CYP21A2* gene was performed at the diagnostic molecular genetic laboratories of university hospitals of the four enrolled cities. The *CYP21A2* gene was screened first for the detection of the eight most common mutations [p.P30L, IVS2-13C>G (IVS-2), p.I172N, exon 6 mutation cluster (p.I236N, p.V237E, p.M239K), p.V281L, p.Q318X, p.R356W, 8-bp-deletion]. Subsequent testing for large deletion and conversion by MLPA or allele specific semi-quantitative PCR/enzyme restriction method and sequencing when needed was performed when indicated.

Consequently, six babies were diagnosed with CAH (four males, two females). Four cases were diagnosed with classical SW 21-OHD (two males, two females), one male baby had SV 21-OHD and one male baby had 11-OHD CAH. None of these babies was premature nor had low birth weight. Diagnosis of CAH was verified by molecular analysis of *CYP21A2* and *CYP11B1* genes in five of the cases (Table 5). The identified mutations in our patients were among the previously known and common mutations analyzed by Sanger sequencing and heterozygosity of parents was confirmed for the identified mutations. There was no report of a case with SW 21-OHD missed during the period of screening in the screening area.

The estimated incidence of classical 21-OHD CAH in the screened population was 1:7,787. In 206 of 38,935 infants there was a false positive recall rate of 0.52%. 

None of the recalled babies with false-positive results had any clinical signs or symptoms suggestive of CAH. The mean±SD duration from birth to clinical evaluation of abnormal screening test results of false-positive cases was 25.8±6.4 days. We have compared first-tier 17-OHP and (21-S+17-OHP)/F values of false-positive recalled babies and babies with 21-OHD. Both of these parameters were significantly higher in babies with 21-OHD compared to term babies and ≥2500 gr birthweight (n=101) with false-positive screening results (Table 6).

## Discussion

The purpose of this prospective pilot study was to estimate the incidence of CAH in Turkey and to assess the characteristics and efficacy of the adopted NBS strategy determine if any modifications to the strategy would enhance screening performance. Newborns were screened for CAH in parallel with the normal Turkish National Newborn Screening Programme in four cities during a six-months time period. Data analysis revealed an estimate of the incidence of classical 21-OHD CAH in the screened population as 1:7,787. Data were analysed to reassess the strategy for the upcoming extended NBS for CAH in Turkey. 

NBS for CAH is universal in the US (15) and many other developed countries (5,6). The incidence of classical CAH is approximately 1:13,000 to 1:15,000 1:14,000 to 1:18,000 in most populations (2,5). However, it is reported to be more prevalent in populations with high rates of consanguinity. The data from NBS for CAH in the United Arab Emirates and Saudi Arabia revealed an incidence of 1:9,030 and 1:7,908, respectively for classical CAH (16,17). Our data demonstrated that incidence of classical CAH in Turkey is similar to that in the Gulf Arab region. This is most probably due to the high rate of consanguinity (overall rate of consanguinity is 22% in Turkey, increasing to 34% in the South East Anatolia region) (18). Therefore, one may expect an increase in the incidence of homozygous biallelic mutations in our population in comparison to compound heterozygotes for two or more different mutant *CYP21A2* alleles. This is indeed the case, three of the five patients identified in the current study were homozygous carriers of biallelic mutations causing classical 21-OHD. Together with the high carrier rate for classical CAH in the general population, which is ~2%, we expect to have a relatively higher incidence of classical CAH in Turkey, which we calculated to be 1:7,787. This is a finding which supports the incorporation of CAH in the core programme of NBS in Turkey.

Screening markedly reduces the time to diagnosis of infants with CAH and will have an impact in reducing serious morbidity and mortality (19,20,21,22). A retrospective analysis of neonatal DBS in the Czech Republic and Austria identified three genotype-proven cases of classical CAH among 242 samples from cases of sudden infant death that were not screened for CAH (23). Previous studies have reported a death rate of ~10% in infants with SW CAH without screening (24), but recent estimates from developed countries are lower, 0-4% (25). We had no mortality due to unrecognized classical CAH among the screened cohort and only one of the cases had severe hyponatremia at the time of diagnosis. However, the fact that there was no mortality so far, does not ensure that the screening strategy is efficient to prevent delay in the diagnosis of CAH cases in the long run is not adding any safety for the screening program in the long run since it is well known that the crisis may occur at one week of age or even earlier. Furthermore, the initiation of hydrocortisone treatment in our study ranged between 10 to 30 days of life in four cases with SW 21-OHD and the mean±SD duration from birth to clinical evaluation of abnormal screening test results of false positive cases was 25.8±6.4 days. In this regard, our pilot study should be criticised for delayed recall of positive screening results. This delayed recall can partially be explained by our single sample second-tier screening approach. A potential disadvantage of single sample second-tier testing or a second specimen programme is that the infant would have been symptomatic by the time of second-tier testing or a second specimen was collected and tested. Therefore, a significantly high 17-OHP value in the first-tier should alarm the clinician for a suspicion of CAH and neonates with elevated 17-OHP levels should be recalled directly. Awaiting a second analysis, even on the same sample may only delay the recall until after the time when the child develops detrimental salt crisis. Furthermore, in cases with a markedly elevated first 17-OHP result, a second tier does not necessarily add important information. Indeed, our first-tier 17-OHP results of 21-OHD CAH babies were very significantly higher than that found in 101 recalled term babies with false positive screening results (302.6±357 ng/mL vs 13.61±4.42 ng/mL, p<0.0001) and in 33967 health term babies with normal first-tier results (3.92±2.43).

Another reason for relatively late recall in our screening programme may be the thrice-weekly postal service of samples from hospitals to screening laboratory and the different location of laboratories for FIA and LC-MS/MS. However, efforts to reduce the recall time are ongoing for the upcoming extended CAH NBS in Turkey by performing two steps of screening in a single central laboratory so that second-tier testing can be performed on the same day that a positive first screening result is obtained. Moreover, the filter paper samples will be collected from hospitals every day and sent to the screening laboratory by regular daily postal service. Nevertheless, since three of five cases with classical 21-OHD diagnosed through our pilot screening were males, and thus without ambiguous genitalia, it is safe to say that the diagnosis and treatment would have been delayed much more without screening. 

Another weakness of single sample second-tier screening is the high false negative rate compared to second sample testing, particularly to diagnose the classical CAH cases with a delayed rise in 17-OHP levels. The Minnesota program has the longest experience in using a single sample two-tier screening algorithm with steroid profiling by LC-MS/MS as the second tier in specimens that exceeded the first-tier 17-OHP cut-off (26). If the second tier test results were negative, further follow-up of the child was considered unnecessary. They have evaluated their 11 years of experience on screening and came to the conclusion that the overall false negative rate doubled with their two-tiered algorithm. This is highlighted by the finding that seven missed cases were not tested by LC-MS/MS because their first-tier 17-OHP values were within range, and four more were missed by the second tier testing after initial abnormal screening values (26). Our single sample, two-tier screening may still have a similar risk to misidentify some CAH cases with delayed rise in 17-OHP. Therefore, physicians should have a high level of suspicion in patients presenting with signs and symptoms of CAH even if they have (false) negative screening results. In view of the high false negative results associated with a single NBS two-tier approach, some programs have opted to collect and screen a second specimen as an alternative means of improving the results of CAH screening. However, this may further confuse the screening results. For example, the Colorado screening program collects the sample in the first two days of life, which may be the main reason for their high false negative rates. Therefore, this program has routinely obtained a second specimen, 1-2 weeks after birth for repeat screening, which was reported to further complicate the screening, due to longer time before recall (27). 

We have also questioned our high recall rate during NBS for CAH in comparison to previous studies (6). Recall rate was reported between 0.002-1.2%, generally <0.5%, in many developed countries with long established screening programs for CAH (6). Furthermore, such low recall rates are reported in the course of single tier DBS screening. We could achieve a recall rate of 0.54%, in the face of a higher cost adopting single DBS-two-tier screening approach. This can be explained by the lower cut-off values we used for the first step 17-OHP FIA measurements as well as second step (21-S+17-OHP)/F ratio to increase the sensitivity of this pilot study. The lower cut-off values have the advantage of increasing screening sensitivity with a markedly increased risk of higher false positive rate, higher cost and higher likelihood of unnecessary treatment of NCCAH cases, and even of non-CAH healthy babies. Further analysis of our data suggests that 99.8^th^ percentile of FIA based 17-OHP levels in our healthy population (excluding the babies with SW 21-OHD) is 50 ng/mL for 1500-2500 gr and 32-36 gw babies and is 20 ng/mL for ≥2500 gr and ≥36 gw babies. When these 17-OHP levels were used as cut-off values in the first-tier we would have expected 253 babies failing to pass, which corresponds to only 11% of population undergoing the second-tier test. Likewise, 161 of 212 babies (75%) had a (21-S+17-OHP)/F ratio of <1 in the second-tier testing while this ratio ranges between 4.6-32.4 in classical SW 21-OHD cases. Even in the single case with SV 21-OHD, the measurement of (21-S+17-OHP)/F ratio was 1.31. Therefore, if we had used 1.0 as the cut-off for (21-S+17-OHP)/F ratio, the recall rate would decrease by 75%. This observation is similar to that of Janzen et al (11) who analyzed the (21-S+17-OHP)/F ratio in around 8000 retrospective and prospective DBS samples in order to compare healthy newborns (including preterms) with 66 CAH cases. None of the cases with CAH had a (21-S+17-OHP)/F ratio <1. Analysis of data from the current study helped us to reassess and modify the screening strategy for the upcoming extended NBS screening for CAH in Turkey. It emerges that the above-mentioned cut-off values may contribute to designing a less labour intensive and more efficient screening strategy for CAH, and also with a better cost-benefit profile.

Immunoassays of DBS for 17-OHP are the most widely used and least costly initial screening methods for CAH. However, poor antibody specificity in addition to abundant cross-reacting hormones in the newborn circulation as well as the necessity of variation in the cut-offs with respect to gestational ages (28) and/or birthweight (29), limit their use in the detection of CAH. Furthermore, stress due to prematurity or critical illness generally increases adrenal F and 17-OHP secretion, which further hamper interpretation of screening results for CAH. Therefore, using 17-OHP as the sole marker may increase the recall rate as well as likelihood of false positive healthy infants who may be started on glucocorticoids unnecessarily. Employing the LC-MS/MS based steroid panel appears as an effective second-tier screen that would better separate false positives, avoid false negatives and potentially save a great deal in unnecessary health care expenditure, which subsequently will relieve much of the stress and work burden experienced by health professionals and parents with quite confusing immunoassay results (11,13,30,31,32). We adopted a modified LC-MS/MS protocol developed by Janzen et al (11) that utilized a ratio of the sum of 17-OHP and 21-S levels, divided by the F level as a second-tier screening test. This protocol was reported to identify all affected children with no false positives for a positive predictive value of 100% (11). Particularly 21-S, which is produced by 11β-hydroxylation of 17-OHP, is not expected to be secreted in large amounts even in preterm infants, and thus elevated levels are highly specific for 21-OHD (33,34).

It is encouraging that simultaneous measurement of 17-OHP, 21-S and F by LC-MS/MS increased the positive predictive value of our CAH screening 9-fold over that for 17-OHP FIA alone. This pilot study also measured 11-S in addition to 17-OHP, 21-S, F and 4AS, which is specifically diagnostic for 11-hydroxylase deficiency. Hence, with our tandem MS method, it was possible to detect a male newborn with (later genotype-proven) classical 11-OHD in addition to cases with 21-OHD. To our knowledge, this is the first patient with 11-OHD identified directly during NBS for CAH. Therefore, the method of steroid profiling has a potential to distinguish other rare forms of classical CAH, beyond 21-OHD, more efficiently. Even though the hormones measured in LC-MS/MS based panels are not specifically diagnostic for the rare forms of CAH such as 11-hydroxylase or 3β-hydroxysteroid dehydrogenase type 2 deficiency, perturbations in simultaneous steroid measurements would provide preliminary information suggesting the need for further evaluation (35,36). In fact, these apparently “rare” forms of classic CAH are far more common in the Middle East and in Turkey, due to the high rate of consanguinity (37,38).

### Study Limitations

The prevelance of 21-OHD was calculated among approximately 40.000 babies screened in 4 pilot cities. This figure may change and may need to be recalculated after an extended newborn screening is completed.

## Conclusion

In conclusion, this pilot study suggests that incidence of CAH in Turkey may be higher than previously reported figures. Hence, it may be recommended that CAH due to 21-OHD be included in the screening panel for the Turkish NBS program. Employing the current LC-MS/MS based steroid panel as second-tier testing may be expected to reduce the time to diagnosis of infants with 21-OHD CAH. It may also enable detection of rare forms of classical CAH. On the other hand, further efforts are needed in our CAH screening programme for earlier clinical recall of babies with positive NBS tests, which is critically important to both prevent salt loss and to shorten the period of unclear sex in classical CAH cases. Prospective analyses of screening strategy, cut-off values and results would help to increase the sensitivity and reduce the false positive rate of screening. Such measures will subsequently serve to alleviate the medical, psychological and economic burden of CAH and its associated health problems.

## Figures and Tables

**Table 1:**
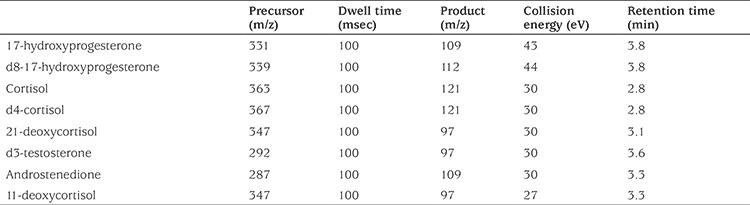
Multiple reaction monitoring functions and settings for detecting steroids by liquid chromatography-tandem mass spectrometry

**Table 2:**
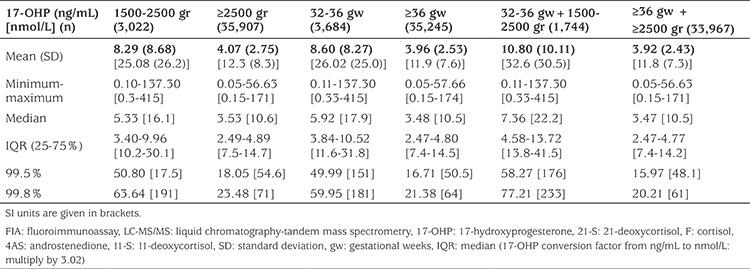
Fluoroimmunoassay based 17-hydroxyprogesterone values of the screened population by birth weight and gestational age

**Table 3:**
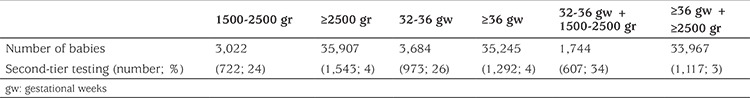
Rate of second-tier testing among babies based on birth weight and gestational weeks

**Table 4:**
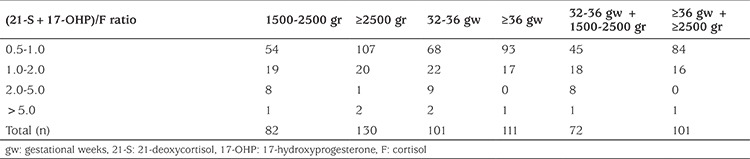
Distribution of babies based on (21-deoxycortisol+17-hydroxyprogesterone)/cortisol ratio adjusted for gestational age and birth weight

**Table 5:**
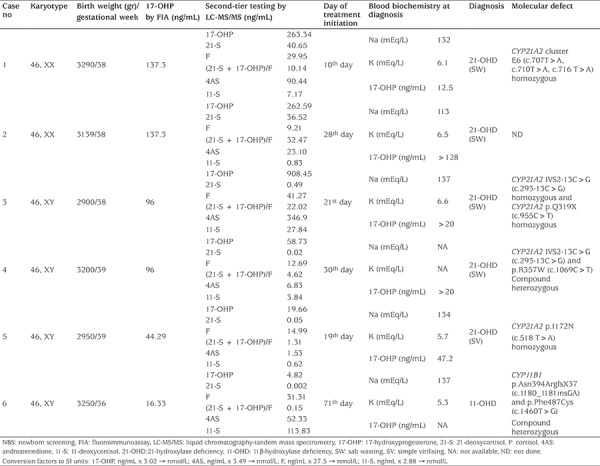
Clinical characteristics and laboratory details of the patients with congenital adrenal hyperplasia diagnosed through newborn screening

**Table 6:**
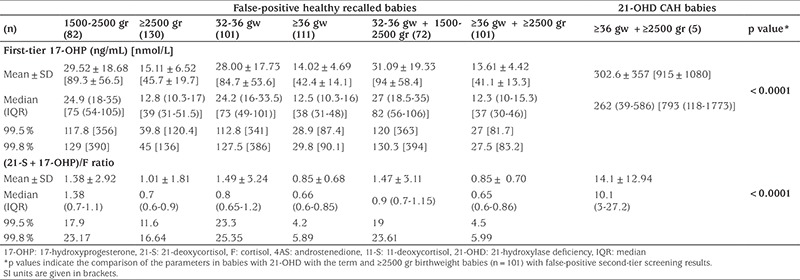
Comparison of first-tier 17-hydroxyprogesterone levels and second-tier (21-deoxycortisol + 17-hydroxyprogesterone)/cortisol ratios between the 206 false-positive healthy recalled infants and 5 infants with 21-hydroxylase deficiency

## References

[1] Hannah-Shmouni F, Chen W, Merke DP (2017). Genetics of Congenital Adrenal Hyperplasia. Endocrinol Metab Clin North Am.

[2] Pang SY, Wallace MA, Hofman L, Thuline HC, Dorche C, Lyon IC, Dobbins RH, Kling S, Fujieda K, Suwa S (1988). Worldwide experience in newborn screening for classical congenital adrenal hyperplasia due to 21-hydroxylase deficiency. Pediatrics.

[3] Clayton PE, Miller WL, Oberfield SE, Ritzén EM, Sippell WG, Speiser PW;, ESPE/ LWPES CAH Working Group (2002). Consensus statement on 21-hydroxylase deficiency from the European Society for Paediatric Endocrinology and the Lawson Wilkins Pediatric Endocrine Society. Horm Res.

[4] Joint LWPES/ESPE CAH Working Group (2002). Consensus statement on 21-hydroxylase deficiency from the Lawson Wilkins Pediatric Endocrine Society and the European Society for Paediatric Endocrinology. J Clin Endocrinol Metab.

[5] Therrell BL, Padilla CD, Loeber JG, Kneisser I, Saadallah A, Borrajo GJ, Adams J (2015). Current status of newborn screening worldwide: 2015. Semin Perinatol.

[6] Gidlöf S, Falhammar H, Thilén A, von Döbeln U, Ritzén M, Wedell A, Nordenström A (2013). One hundred years of congenital adrenal hyperplasia in Sweden: a retrospective, population-based cohort study. Lancet Diabetes Endocrinol.

[7] Dumic K, Krnic N, Skrabic V, Stipancic G, Cvijovic K, Kusec V, Stingl K (2009). Classical congenital adrenal hyperplasia due to 21-hydroxylase deficiency in croatia between 1995 and 2006. Horm Res.

[8] Working Group on Neonatal Screening of the European Society for Paediatric Endocrinology (2001). Procedure for neonatal screening for congenital adrenal hyperplasia due to 21-hydroxylase deficiency. Horm Res.

[9] Cavarzere P, Camilot M, Teofoli F, Tatò L (2005). Neonatal screening for congenital adrenal hyperplasia in North-Eastern Italy: a report three years into the program. Horm Res.

[10] Lee JE, Moon Y, Lee MH, Jun YH, Oh KI, Choi JW (2008). Corrected 17-alphahydroxyprogesterone values adjusted by a scoring system for screening congenital adrenal hyperplasia in premature infants. Ann Clin Lab Sci.

[11] Janzen N, Peter M, Sander S, Steuerwald U, Terhardt M, Holtkamp U, Sander J (2007). Newborn screening for congenital adrenal hyperplasia: additional steroid profile using liquid chromatography-tandem mass spectrometry. J Clin Endocrinol Metab.

[12] Kim B, Lee MN, Park HD, Kim JW, Chang YS, Park WS, Lee SY (2015). Dried blood spot testing for seven steroids using liquid chromatographytandem mass spectrometry with reference interval determination in the Korean population. Ann Lab Med.

[13] Boelen A, Ruiter AF, Claahsen-van der Grinten HL, Endert E, Ackermans MT (2016). Determination of a steroid profile in heel prick blood using LC-MS/ MS. Bioanalysis.

[14] Hayashi G, Faure C, Brondi MF, Vallejos C, Soares D, Oliveira E, Brito VN, Mendonca BB, Bachega TA (2011). Weight-adjusted neonatal 17-OHprogesterone cutoff levels improve the efficiency of newborn screening for congenital adrenal hyperplasia. Arq Bras Endocrinol Metabol.

[15] National Newborn Screening and Global Resource Center. 2006. National Newborn Screening System [Online]. National Newborn Screening & Global Resource Center.

[16] Al Hosani H, Salah M, Osman HM, Farag HM, El-Assiouty L, Saade D, Hertecant J (2014). Expanding the comprehensive national neonatal screening programme in the United Arab Emirates from 1995 to 2011. East Mediterr Health J.

[17] Alfadhel M, Al Othaim A, Al Saif S, Al Mutairi F, Alsayed M, Rahbeeni Z, Alzaidan H, Alowain M, Al-Hassnan Z, Saeedi M, Aljohery S, Alasmari A, Faqeih E, Alwakeel M, AlMashary M, Almohameed S, Alzahrani M, Migdad A, Al-Dirbashi OY, Rashed M, Alamoudi M, Jacob M, Alahaidib L, El-Badaoui F, Saadallah A, Alsulaiman A, Eyaid W, Al-Odaib A (2017). Expanded Newborn Screening Program in Saudi Arabia: Incidence of screened disorders. J Paediatr Child Health.

[18] Koc I (2008). Prevalence and sociodemographic correlates of consanguineous marriages in Turkey. J Biosoc Sci.

[19] Balsamo A, Cacciari E, Piazzi S, Cassio A, Bozza D, Pirazzoli P, Zappulla F (1996). Congenital adrenal hyperplasia: neonatal mass screening compared with clinical diagnosis only in the Emilia-Romagna region of Italy, 1980-1995. Pediatrics.

[20] Brosnan PG, Brosnan CA, Kemp SF, Domek DB, Jelley DH, Blackett PR, Riley WJ (1999). Effect of newborn screening for congenital adrenal hyperplasia. Arch Pediatr Adolesc Med.

[21] Therrell BL Jr, Berenbaum SA, Manter-Kapanke V, Simmank J, Korman K, Prentice L, Gonzalez J, Gunn S (1998). Results of screening 1.9 million Texas newborns for 21-hydroxylase-deficient congenital adrenal hyperplasia. Pediatrics.

[22] Thil’en A, Nordenström A, Hagenfeldt L, von Döbeln U, Guthenberg C, Larsson A (1998). Benefits of neonatal screening for congenital adrenal hyperplasia (21-hydroxylase deficiency) in Sweden. Pediatrics.

[23] Strnadová KA, Votava F, Lebl J, Mühl A, Item C, Bodamer OA, Torresani T, Bouska I, Waldhauser F, Sperl W (2007). Prevalence of congenital adrenal hyperplasia among sudden infant death in the Czech Republic and Austria. Eur J Pediatr.

[24] Watson MS, Mann MY, Lloyd-Puryear MA, Rinaldo P, Howell RR (2006). Newborn screening: toward a uniform screening panel and system. Genet Med.

[25] Grosse SD, Van Vliet G (2007). How many deaths can be prevented by newborn screening for congenital adrenal hyperplasia?. Horm Res.

[26] Sarafoglou K, Banks K, Gaviglio A, Hietala A, McCann M, Thomas W (2012). Comparison of one-tier and two-tier newborn screening metrics for congenital adrenal hyperplasia. Pediatrics.

[27] Chan CL, McFann K, Taylor L, Wright D, Zeitler PS, Barker JM (2013). Congenital adrenal hyperplasia and the second newborn screen. J Pediatr.

[28] Gruneiro-Papendieck L, Prieto L, Chiesa A, Bengolea S, Bossi G, Bergada C (2001). Neonatal screening program for congenital adrenal hyperplasia: adjustments to the recall protocol. Horm Res.

[29] Allen DB, Hoffman GL, Fitzpatrick P, Laessig R, Maby S, Slyper A (1997). Improved precision of newborn screening for congenital adrenal hyperplasia using weight-adjusted criteria for 17-hydroxyprogesterone levels. J Pediatr.

[30] Seo JY, Park HD, Kim JW, Oh HJ, Yang JS, Chang YS, Park WS, Lee SY (2014). Steroid profiling for congenital adrenal hyperplasia by tandem mass spectrometry as a second-tier test reduces follow-up burdens in a tertiary care hospital: a retrospective and prospective evaluation. J Perinat Med.

[31] Lacey JM, Minutti CZ, Magera MJ, Tauscher AL, Casetta B, McCann M, Lymp J, Hahn SH, Rinaldo P, Matern D (2004). Improved specificity of newborn screening for congenital adrenal hyperplasia by second-tier steroid profiling using tandem mass spectrometry. Clin Chem.

[32] Kim B, Lee MN, Park HD, Kim JW, Chang YS, Park WS, Lee SY (2015). Dried blood spot testing for seven steroids using liquid chromatographytandem mass spectrometry with reference interval determination in the Korean population. Ann Lab Med.

[33] Fiet J, Villette JM, Galons H, Boudou P, Burthier JM, Hardy N, Soliman H, Julien R, Vexiau P, Gourmelen M, Kuttenn F (1994). The application of a new highlysensitive radioimmunoassay for plasma 21-deoxycortisol to the detection of steroid-21-hydroxylase deficiency. Ann Clin Biochem.

[34] Cristoni S, Cuccato D, Sciannamblo M, Bernardi LR, Biunno I, Gerthoux P, Russo G, Weber G, Mora S (2004). Analysis of 21-deoxycortisol, a marker of congenital adrenal hyperplasia, in blood by atmospheric pressure chemical ionization and electrospray ionization using multiple reaction monitoring. Rapid Commun Mass Spectrom.

[35] Krone N, Grötzinger J, Holterhus PM, Sippell WG, Schwarz HP, Riepe FG (2009). Congenital adrenal hyperplasia due to 11-hydroxylase deficiency-- insights from two novel CYP11B1 mutations (p. M92X, p.R453Q). Horm Res.

[36] Araújo VG, Oliveira RS, Gameleira KP, Cruz CB, Lofrano-Porto A (2014). 3β-hydroxysteroid dehydrogenase type II deficiency on newborn screening test. Arq Bras Endocrinol Metabol.

[37] Khattab A, Haider S, Kumar A, Dhawan S, Alam D, Romero R, Burns J, Li D, Estatico J, Rahi S, Fatima S, Alzahrani A, Hafez M, Musa N, Razzghy Azar M, Khaloul N, Gribaa M, Saad A, Charfeddine IB, Bilharinho de Mendonça B, Belgorosky A, Dumic K, Dumic M, Aisenberg J, Kandemir N, Alikasifoglu A, Ozon A, Gonc N, Cheng T, Kuhnle-Krahl U, Cappa M, Holterhus PM, Nour MA, Pacaud D, Holtzman A, Li S, Zaidi M, Yuen T, New MI (2017). Clinical, genetic, and structural basis of congenital adrenal hyperplasia due to 11β-hydroxylase deficiency. Proc Natl Acad Sci U S A.

[38] Kandemir N, Yilmaz DY, Gonc EN, Ozon A, Alikasifoglu A, Dursun A, Ozgul RK (2017). Novel and prevalent CYP11B1 gene mutations in Turkish patients with 11-β hydroxylase deficiency. J Steroid Biochem Mol Biol.

